# Reconstitution of CD8 T Cells Protective against Cytomegalovirus in a Mouse Model of Hematopoietic Cell Transplantation: Dynamics and Inessentiality of Epitope Immunodominance

**DOI:** 10.3389/fimmu.2016.00232

**Published:** 2016-06-14

**Authors:** Rafaela Holtappels, Niels A. W. Lemmermann, Jürgen Podlech, Stefan Ebert, Matthias J. Reddehase

**Affiliations:** ^1^Institute for Virology and Research Center for Immunotherapy (FZI), University Medical Center of the Johannes Gutenberg-University Mainz, Mainz, Germany

**Keywords:** adoptive cell transfer, CD8 T cells, cytomegalovirus, epitope prediction, hematopoietic cell transplantation, immunodominance, immunotherapy, reconstitution

## Abstract

Successful reconstitution of cytomegalovirus (CMV)-specific CD8^+^ T cells by hematopoietic cell transplantation (HCT) gives a favorable prognosis for the control of CMV reactivation and prevention of CMV disease after hematoablative therapy of hematopoietic malignancies. In the transient immunocompromised state after HCT, pre-emptive cytoimmunotherapy with viral epitope-specific effector or memory CD8^+^ T cells is a promising option to speed up antiviral control. Despite high-coding capacity of CMVs and a broad CD8^+^ T-cell response on the population level, which reflects polymorphism in major histocompatibility complex class-I (MHC-I) glycoproteins, the response in terms of quantity of CD8^+^ T cells in any individual is directed against a limited set of CMV-encoded epitopes selected for presentation by the private repertoire of MHC-I molecules. Such epitopes are known as “immunodominant” epitopes (IDEs). Besides host immunogenetics, genetic variance in CMV strains harbored as latent viruses by an individual HCT recipient can also determine the set of IDEs, which complicates a “personalized immunotherapy.” It is, therefore, an important question if IDE-specific CD8^+^ T-cell reconstitution after HCT is critical or dispensable for antiviral control. As viruses with targeted mutations of IDEs cannot be experimentally tested in HCT patients, we employed the well-established mouse model of HCT. Notably, control of murine CMV (mCMV) after HCT was comparably efficient for IDE-deletion mutant mCMV-Δ4IDE and the corresponding IDE-expressing revertant virus mCMV-Δ4IDE-rev. Thus, antigenicity-loss mutations in IDEs do not result in loss-of-function of a polyclonal CD8^+^ T-cell population. Although IDE deletion was not associated with global changes in the response to non-IDE epitopes, the collective of non-IDE-specific CD8^+^ T-cells infiltrates infected tissue and confines infection within nodular inflammatory foci. We conclude from the model, and predict also for human CMV, that there is no need to exclusively aim for IDE-specific immunoreconstitution.

## Introduction

Reactivation of human cytomegalovirus (hCMV), the prototype member of the β-subfamily of herpesviruses, continues to be a medical challenge in recipients of solid organ transplantation (SOT) as well as in the therapy of hematopoietic malignancies by hematoablative treatment followed by hematopoietic cell transplantation (HCT) for reconstitution. In both cases, latent CMV is released from immune surveillance by a therapy-inherent immunosuppressive regimen, host-versus-graft reaction (graft rejection) prophylaxis in SOT, and hematoablative treatment as well as graft-versus-host disease prophylaxis in HCT. Notably, risk of graft failure after SOT is primarily associated with donor seropositivity for anti-hCMV antibodies (D^+^), which is indicative of latent infection of the transplant donor, whereas in HCT risk of multiple-organ CMV disease is associated mainly with recipient seropositivity (R^+^) indicative of latent infection of the recipient [for reviews see Ref. (1–6)]. Although myeloid lineage hematopoietic progenitor cells are a recognized reservoir for latent hCMV and, thus, a potential source of reactivated virus [(7–11); for reviews see Ref. (12, 13)], the risk association in both SOT and HCT may point to latently infected non-hematopoietic stromal or parenchymal tissue cells as the primary source(s) of reactivated virus causing CMV organ disease. The model of murine CMV (mCMV) infection reflected the clinical correlate in that reactivation-competent latent virus was transmitted by experimental SOT (14–16) and failed to be transmitted by experimental HCT (17) but reactivated independently from multiple organs (18–21). Specifically, liver sinusoidal endothelial cells (LSECs) proved to be a cellular site of mCMV latency and reactivation (22), and in explants of latently infected lung tissue, virus reactivation failed to colocalize with CD11b^+^ and CX3CR1^+^ cells of the hematopoietic myeloid differentiation lineage (23).

In clinical HCT with latently infected patients as recipients (R^+^), most cases of CMV disease are diagnosed 3–12 weeks after HCT [reviewed in Ref. (5)]. Thus, considering that minute amounts of reactivated virus need time to substantially multiply and spread before CMV disease can be diagnosed, reactivation apparently occurs rapidly after hematoablative treatment. Since reactivation is a stochastic and, thus, unpredictable event [(24, 25), reviewed in Ref. (26–28)], murine models mimic a 100% “reactivation rate” by experimental primary infection shortly after experimental HCT for an easier statistical evaluation.

Early studies on immune reconstitution and CMV disease after clinical HCT have identified reconstitution of cytotoxic responses as a positive predictor for prevention of CMV disease (29). These findings were corroborated and further specified for recipients of HLA-matched clinical HCT by demonstrating that CMV pneumonia did not occur in patients in whom CD8^+^ HLA class-I restricted, hCMV-specific cytolytic T lymphocytes (CTL) developed in the course of hematopoietic reconstitution (30). While these clinical studies gave good correlative evidence for prevention of CMV disease by CMV-specific CD8^+^ T cells, pre-emptive cytoimmunotherapy of CMV disease by adoptive transfer of purified polyclonal *ex vivo* populations or of virus epitope-specific clonal and non-clonal CTL lines (CTLL) or sorted CD8^+^ T cells provided “proof of concept” for antiviral protection by CD8^+^ T cells [reviewed in Ref. (31–34)]. This was pioneered by the mouse model (35, 36) and later confirmed in clinical trials (37–41). Supplementation of HCT with CMV-specific CD8^+^ T cells revealed that combined endogenous and adoptive reconstitution of antiviral CD8^+^ T cells prevents lethal CMV disease, limits latent virus burden, and reduces the risk of virus recurrence for late CMV disease in HCT recipients in the murine model (42). More recently, protective antiviral function of human CD8^+^ T cells specific for an hCMV UL83/pp65-derived peptide was also shown in an HLA-A2 transgenic mouse model upon challenge infection with a “humanized” mCMV recombinant expressing the hCMV epitope (43).

Inevitable death from multiple-organ CMV disease after HCT following depletion of pan-CD8^+^, but not of pan-CD4^+^ T cells, revealed that CD8^+^ effector cell function is essential for preventing CMV disease after HCT and excluded redundant control by innate or by other adaptive immune effector cell types [(44, 45), see also the accompanying Review article in this issue of *Frontiers in Immunology*]. The contribution of different viral epitopes to the protective reconstitution after HCT, however, is less well established.

Although hCMV and mCMV both have a high-coding capacity with the potential to encode numerous antigenic peptides presented by major histocompatibility complex class-I (MHC-I) molecules to the T-cell receptors (TCRs) of CD8^+^ T cells, the response in any individual person (46) as well as in mouse inbred strains (31–33, 47) is limited to only few epitopes selected by the individual HLA/MHC class-I genotype. The response is broad on the population level, accounting for HLA/MHC polymorphism, but is narrow in any individual. Moreover, among the set of epitopes, only few give rise also to an easily detectable, numerically prominent *in vivo* response and are, thus, operationally classified as being “immunodominant” in terms of quantity. UL83/pp65 is the prototypic example of an hCMV protein that primes and expands a high proportion of CD8^+^ T cells [(48–51), reviewed in Ref. (52)], and in the mouse model an H-2L^d^-presented m123/IE1-derived peptide is the prototype of an “IDE” [(53, 54), reviewed in Ref. (31)]. Although it was tempting to select such epitopes for adoptive immunotherapy or vaccine design, “immunodominance” in quantity is not necessarily identical with “immunodominance” in protective function. Specifically, in the mouse model, adoptive transfer of epitope-specific CTLL revealed an equally efficient antiviral protection with “subdominant” epitopes [reviewed in Ref. (32–34)], a finding corroborated by DNA vaccination based on “subdominant” epitopes (55). In accordance with this, deletion of “IDEs” did not reduce the protective efficacy of mCMV-primed polyclonal CD8^+^ T cells upon adoptive transfer, regardless of whether these epitopes were missing in the cell transfer donor, the recipient, or both (56, 57).

In the cell transfer models, effector and memory cells, primed from naïve CD8^+^ T cells following CMV infection of an immunocompetent host, were used for testing their antiviral function. This is not necessarily predictive for the protective contribution of “immunodominant” and “subdominant” viral epitopes after HCT when CD8^+^ T cells are derived from hematopoietic lineage reconstitution and thymic selection in the presence of CMV. Here, we have analyzed the mCMV epitope-specific reconstitution of antiviral CD8^+^ T cells over time after syngeneic experimental HCT and addressed the question if deletion of all known “IDEs” has a loss-of-control phenotype comparable to pan-CD8^+^ T-cell depletion.

## Materials and Methods

### Prediction Algorithms for Processing Scores and Statistical Analyses

Processing predictions (proteasomal cleavage/transporter associated with antigen processing/presentation (TAP)/MHC class-I combined predictor) were made using the IEDB analysis resource (*http://tools.iedb.org/processing/)*. This tool predicts epitope candidates based on the processing of peptides in the cell. It combines predictors for proteasomal processing by the constitutive proteasome or the immunoproteasome (58), TAP transport (59), and MHC-I binding (60) to produce an overall score for each peptide’s intrinsic potential of being a T-cell epitope. The MHC-I binding predictions were made on 3/08/2016 using the IEDB analysis resource Consensus tool (61), which combines predictions from ANN aka NetMHC 3.4 (62, 63), SMM (64), and Comblib (65).

The two-sided unpaired *t*-test with Welch’s correction of unequal variances was used to evaluate statistical significance of differences between two independent sets of log-transformed data. Differences were considered statistically significant for *P*-values of <0.05 (*) or highly significant for *P*-values of <0.001 (**). Viral doubling times (vDT = log2/*a)* in host organs and the corresponding 95% confidence intervals were calculated by linear regression analysis from the slopes *a* of log-linear growth curves (66). Frequencies of cells responding in the enzyme-linked immunospot (ELISpot) assay and the corresponding 95% confidence intervals were calculated by intercept-free linear regression analysis from the linear portions of regression lines based on spot counts from triplicate assay cultures for each of the graded cell numbers seeded. Calculations were performed with GraphPad Prism 6.04 (GraphPad Software, San Diego, CA, USA).

### Hematopoietic Cell Transplantation, Adoptive Cell Transfer, and Infection

Technical details of HCT (67), the immunomagnetic enrichment (positive selection) of CD8^+^ T cells for adoptive transfer or for ELISpot assays (66), intravenous adoptive cell transfer/cytoimmunotherapy (66), and intraplantar (footpad) infection (66) procedures were described by us previously in method book chapters. In brief, recipients of HCT as well as of T-cell transfer were 8-week-old female BALB/c (*H-2^*d*^* haplotype) mice, immunocompromised by hematoablative total-body γ irradiation with a single dose of 6.5 Gy. Cells were infused intravenously, usually 4 h after the hematoablative conditioning. For syngeneic HCT with BALB/c mice as donors and recipients, 5 × 10^6^ donor-derived femoral and tibial bone marrow cells (BMC), depleted of CD8^+^ cells by immunomagnetic separation, were infused. For adoptive cell transfer, CD8^+^ (memory) T cells were immunomagnetically purified from spleen cells of BALB/c donor mice primed to mCMV antigens by intraplantar infection at >3 months before use. Graded cell numbers were infused intravenously into corresponding groups of usually five immunocompromised recipients each. Intraplantar infection of the recipients of HCT or adoptive cell transfer was performed shortly thereafter with 10^5^ plaque-forming units (PFU) of the viruses mCMV-Smith [ATCC VR-194; derived from batch 9/75; (68)], mCMV-BAC^W^ [cloned bacterial artificial chromosome-derived virus MW97.01; (69)], BAC^W^-derived mutant virus mCMV-Δ4IDE (57) or revertant virus mCMV-Δ4IDE-rev (70).

Mice were bred and kept under specified pathogen-free conditions in the Central Laboratory Animal Facility (CLAF) at the University Medical Center of the Johannes Gutenberg-University Mainz.

### Assays for *Ex Vivo* Detection and Quantitation of Viral Epitope-Specific CD8^+^ T Cells

ELISpot assays (each spot representing one responding cell), based on interferon (IFN)-γ secretion by primed viral epitope-specific CD8^+^ T cells upon restimulation with synthetic antigenic peptides, were performed for graded responder cell numbers and a fixed number of peptide-loaded stimulator cells (P815 mastocytoma cells, *H-2^*d*^*) in triplicate assay cultures as described in greater detail previously [(71), and references therein]. Lung-resident CD8^+^ T cells, which include cells in interstitial infiltrates and non-circulating cells trapped in the lung vasculature attached to the endothelium, were isolated in a kinetics post-HCT and infection with mCMV-Smith. Lungs were perfused but bronchoalveolar lavage was not performed in order to discard cells circulating in the blood of the lung vasculature but to retrieve cells localizing to the alveolar epithelium (67, 72, 73).

Synthetic peptides corresponding to reported epitopes presented by MHC-I molecules K^d^, D^d^, and L^d^ were from open reading frames (ORFs) m04 (74), m18 (75), M45 (76), M83 (77), M84 (78), M105 (56), m123/IE1 (53), m145 (56), and m164 (79) (Table 1).

**Table 1:** **List of currently identified CD8^+^ T cell epitopes in the *H-2^d^* haplotype**.

ORF	Antigenic peptide	Presenting MHC-I	Classification	Reference
m04	^243^YGPSLYRRF^251^	D^d^	Subdominant	(74)
m18	^346^SGPSRGRII^354^	D^d^	Subdominant	(75)
M45	^507^VGPALGRGL^515^	D^d^	Subdominant	(76)
M54	^83^RGPYSDEL^90^	D^d^	Subdominant	(70)
M83	^761^YPSKEPFNF^769^	L^d^	Subdominant	(77)
M84	^297^AYAGLFTPL^305^	K^d^	Subdominant	(78)
M105	^207^TYWPVVSDI^215^	K^d^	Dominant	(56)
m123/IE1	^168^YPHFMPTNL^176^	L^d^	Dominant	(53)
m145	^451^CYYASRTKL^459^	K^d^	Dominant	(56)
m164	^150^AGPPRYSRI^158^	D^d^	Dominant	(79, 89)

For detecting also mCMV antigen-specific CD8^+^ T cells for which the epitope is not yet identified, a viral genome-wide ORF library of transfectants [for the principle of the ORF library screening, see Ref. (34, 47)] was used for stimulating memory spleen cells (pool from at least five mice per group) derived from HCT recipients in the phase of latency after clearance of productive infection with viruses mCMV-Δ4IDE and mCMV-Δ4IDE-rev. After electronic gating on CD8^+^ T cells, sensitized cells were quantitated by cytofluorometric detection of intracellular IFN-γ as described in detail previously (66). For control, the same spleen cell populations were tested with the cytofluorometric intracellular IFN-γ assay after stimulation with synthetic peptides corresponding to the set of known epitopes. A list allocating ORF library numbers to actual ORFs can be found in Table S1 in Supplementary Material of Ref. (56).

### Assays for Quantitating Peptide Processing and for Detecting Cell Surface Presentation

Naturally processed antigenic peptides, protected by MHC-I binding against degradation, were isolated from extracts of infected mouse embryo fibroblasts (MEF). They were quantitated by a bioassay with epitope-specific CTLL, generated as described previously (66), and the corresponding synthetic peptides for the standard curve (76, 80), applying methods essentially based on reference (81).

Presentation of peptide–MHC-I (pMHC-I) complexes at the cell surface of infected MEF was detected with the corresponding epitope-specific CTLL in the IFN-γ-based ELISpot assay (see above, except that infected MEF were used as stimulator cells). MEF infection was performed with 0.2 PFU per cell under conditions of “centrifugal enhancement of infectivity” (67), which results in an effective multiplicity of infection (MOI) of 4. For mapping presentation to the kinetic phase of the viral gene expression program, MEF were metabolically arrested in the “immediate-early” (IE) and “early” (E) phases, as described (67), or were allowed to proceed to the “late” (L) phase in absence of inhibitors.

### Assays for Quantitating *In Vivo* Infection and Formation of Nodular Inflammatory Foci

Infectious virus in host organs was quantitated in terms of PFU by testing organ homogenates in a virus plaque assay on MEF under conditions of “centrifugal enhancement of infectivity” (see above), as described in greater detail previously (67).

To evaluate and quantitate infection in the microanatomical context of host tissues, specifically of liver tissue, imaging of infected cells in tissue sections was performed by immunohistochemical (IHC) staining of intranuclear IE1 protein. For detecting nodular inflammatory foci (NIF), a two-color IHC was performed combining the staining of infected cells expressing intranuclear IE1 protein and the staining of T cells expressing CD3ϵ at the cell membrane. IHC methods are described in greater detail in method book articles (66, 67).

## Results

### High Dynamics and Variance of Epitope-Specific CD8^+^ T-Cell Reconstitution after HCT

CD8^+^ T-cell responses are usually characterized by a peak of the primary response during which naïve CD44^low^CD62L^high^ T cells are primed to yield CD44^high^CD62L^low^KLRG1^high^ cytolytic effector cells (T_E_) as well as memory cells (T_M_), subdivided into CD44^high^CD62L^low^KLRG1^low^ effector-memory cells (T_EM_) and CD44^high^CD62L^high^KLRG1^low^ central memory cells (T_CM_). This acute response is followed by a phase of contraction to a primarily lymphoid tissue-resident pool of T_CM_ that can initiate a recall response with recruitment to extra-lymphoid sites of antigen presentation [reviewed in Ref. (82)]. In difference to this general scheme, the CD8^+^ T-cell response to CMVs is characterized by a contraction phase being followed by a more or less continuous increase in the pool size also of extra-lymphoid CD8^+^CD62L^low^ T cells specific for certain viral epitopes. This phenomenon was first described in the HCT model of mCMV infection for pulmonary CD8^+^CD62L^low^ cells, then referred to as T_EM_, specific for two *H-2^*d*^*-restricted epitopes in the BALB/c mouse strain (79, 83). It was later extended to non-HCT models, other organ sites, and to the *H-2^*b*^* haplotype (84), and was coined with the term “memory inflation (MI)” (85). The “inflationary” cells assume the phenotype of short-lived effector cells [SLECs (82)], namely CD44^high^CD62L^low^CD127^low^KLRG1^high^ (86). It is current view that MI is related to viral latency [reviewed in Ref. (27, 28)] and is driven in the intravascular compartment (73) by sporadic viral gene expression events in latently infected non-hematopoietic cells (87, 88), such as endothelial cells, including the LSECs in the liver sinusoids, a defined cellular site of mCMV latency and reactivation (22).

We have here retrospectively compiled data on epitope-specific (for a list of currently defined mCMV epitopes in the *H-2^*d*^* haplotype, see Table 1) CD8^+^ T-cell reconstitution and recruitment into the pulmonary pool from three experimental HCTs performed independently over the years under nominally identical experimental conditions, including identical age of the HCT recipients as an important parameter of expectable thymic T-cell output.

**Figure 1:**
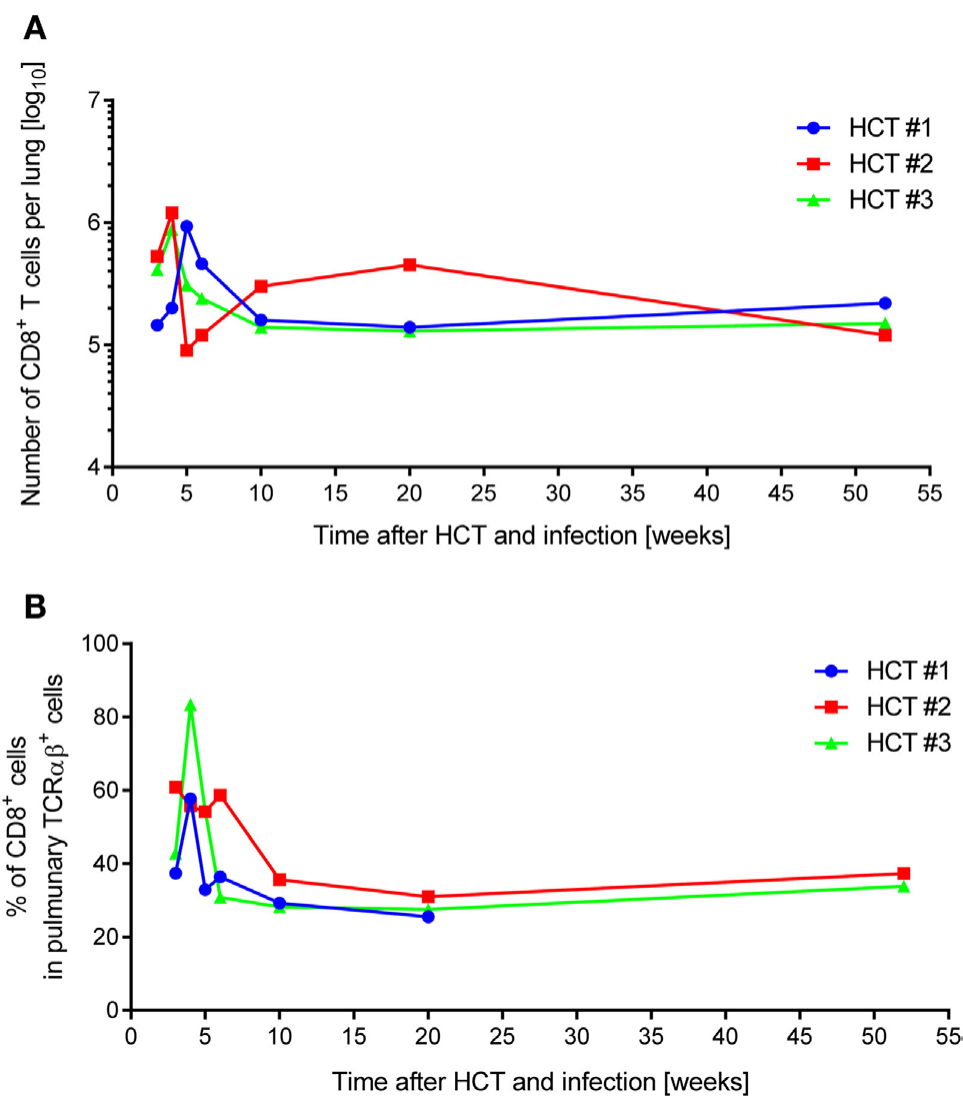
**Dynamics of pulmonary CD8^+^ T-cell infiltration after HCT and infection**. One-year follow-up of CD8^+^ T-cell reconstitution and pulmonary infiltration in mCMV-Smith-infected BALB/c HCT recipients for three independent HCTs (HCT #1, #2, and #3) performed under identical conditions. **(A)** Average total yield of immunomagnetically purified CD8^+^ T lymphocytes per lung isolated from a pool of lungs of at least four HCT recipients per time point. **(B)** Proportion of CD8^+^ T lymphocytes among pulmonary TCRα/β^+^ T cells, determined by cytofluorometric analysis of isolated lung infiltrate cells.

In absolute terms, the yield of pulmonary infiltrate CD8^+^ T cells (Figure 1A) peaked during acute lung infection in weeks 4–5 at ca. 10^6^ cells in all three HCTs, which is in good accordance with previous findings in this model [(54), see also the accompanying Review article in this issue of *Frontiers in Immunology*]. Also in agreement with previous findings is the subsequent decline in the absolute numbers, corresponding with cleared productive infection and establishment of latent infection, but also the long-term persistence of an elevated level of tissue-resident CD8^+^ T cells compared to a control HCT with no infection (45, 83). Notably, as shown previously, long-term tissue-resident CD8^+^ T cells are not exhausted but are functional in that they can control infection upon adoptive transfer into infected immunocompromised recipients (45). While HCT #1 and HCT #3 were similar in the time course, HCT #2 showed a striking discontinuity in that the peak was followed by a rapid 1-log decline and slow recovery until values returned to the “viral latency-associated” level of the two other HCTs. We do not know what an event has caused such a rapid sinking in the lungs in HCT #2, possibly a non-diagnosed other infection detracting the cells from the lungs to other locations, but one must be aware of the fact that unpredictable events may happen also in HCT patients, who all have individual histories of malignant disease and treatment, and of unknown antigen encounters in the phase of reconstitution.

Whereas absolute numbers are prone to variance in isolation efficiency, proportions are less influenced by technical parameters. Similar as described previously for this model (54), CD8^+^ T cells dominated the pulmonary TCRα/β T-cell infiltrates during acute lung infection in all three HCTs (Figure 1B). In this respect, HCT #3, rather than HCT #2 (see above), was exceptional in that a particularly high proportion of CD8^+^ T cells at week 4 dropped markedly within a week.

All in all, independently performed but otherwise identical HCTs showed a similar course of CD8^+^ T-cell reconstitution and lung infiltration in the more cardinal parameters of peak tissue infiltration during acute lung infection, followed by decline and long-term tissue-residency during latent infection of the lungs, but with occasional short-term fluctuations.

**Figure 2:**
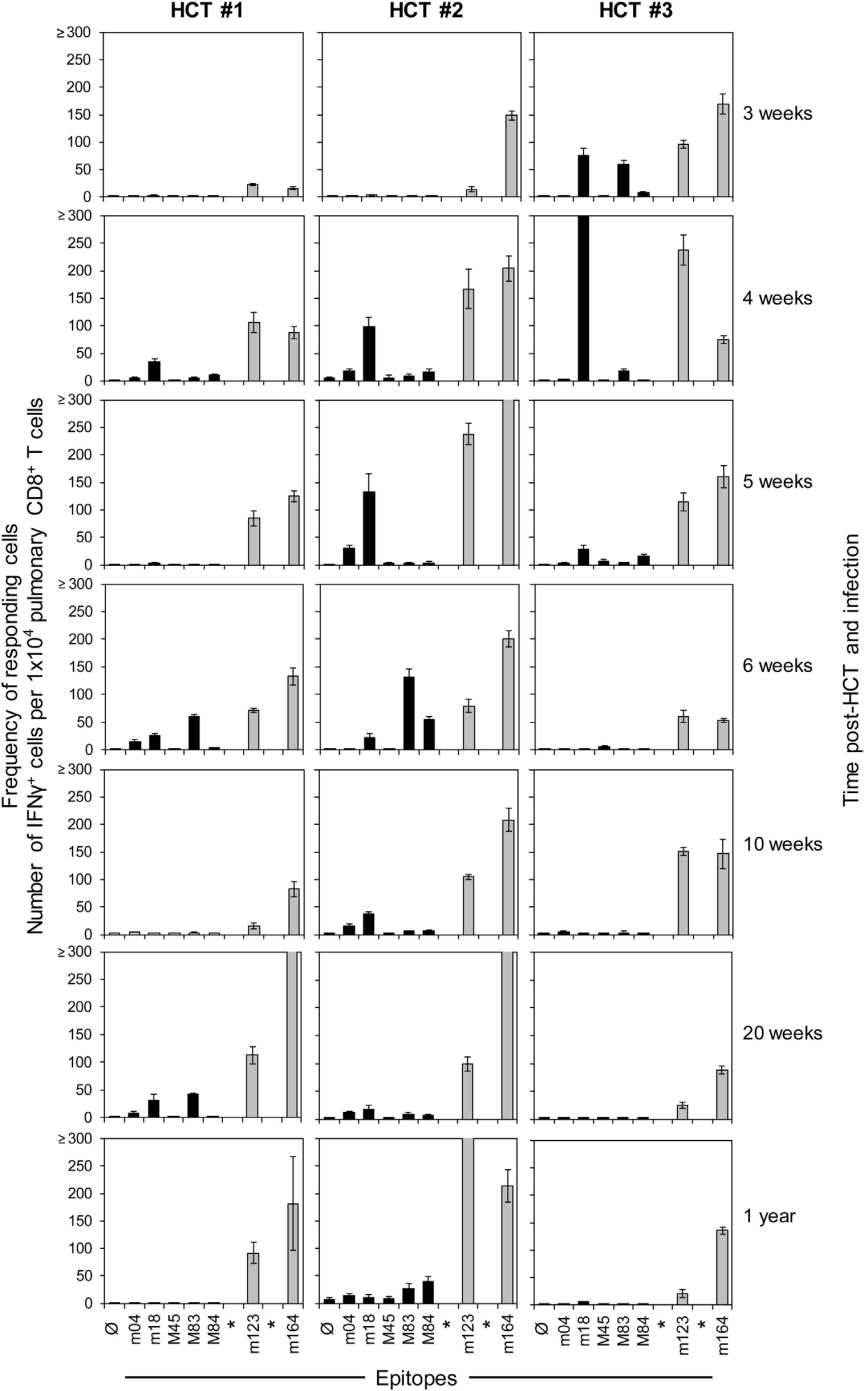
**High dynamics of epitope-recognition patterns after HCT**. Corresponding to Figure 1, frequencies of immunomagnetically purified pulmonary CD8^+^ T cells responding in an ELISpot assay with IFN-γ secretion to stimulation with peptide-loaded stimulator cells were determined for a pool of at least four mice per indicated time. Bars represent the most probable numbers, and error bars indicate the 95% confidence intervals determined by linear regression analysis. Gray-shaded bars highlight responses to epitopes known to drive memory inflation (MI), namely m123/IE1 and m164. *Epitopes addressed later in this work but not yet known at the time when these HCTs were performed. Ø: no peptide added.

In order to monitor for viral epitope-specific pulmonary CD8^+^ T-cell expansions and contractions, we determined the response hierarchy profiles defined by frequencies of CD8^+^ T cells responding to the presentation of a panel of antigenic peptides (Figure 2). In essence, for an observation period of 1 year, epitope-specific responses were highly variable between the three HCTs and far from continuity within any of the three HCTs. For the response to IDEs m123/IE1 and m164, both of which are expressed in the IE phase of the viral gene expression program (53, 89), the time of the peak acute response was variable and contraction was not pronounced. One may discuss contraction at 10 and 6 weeks in HCT #1 and HCT #3, respectively, but not in HCT #2. However, after the establishment of viral latency, which under conditions of HCT is beyond 4 months (87), the established “MI epitopes” m123/IE1 and m164 dominated in accordance with previous findings (79). Note that two additional IDEs, namely M105 and m145 (56), were not yet known when the three HCTs were performed. Likewise, non-IDE M54 has been identified only recently (70). Strikingly, certain epitopes classified in immunocompetent mice as “subdominant” or non-IDEs, transiently popped up, for instance, m18 (weeks 4, 6, and 20) and M83 (weeks 6 and 20) in HCT #1, m18 (weeks 4 and 5) and M83 as well as M84 (week 6, and to a lower level after 1 year) in HCT #2, and m18 (weeks 3 and 4, and declined in week 5) and M83 (week 3) in HCT #3. While m18, M83, and M84 were seen repeatedly and in all three HCTs, the time of their response involvement was highly variable. Most notably, in HCT #3, CD8^+^ T cells specific for the non-IDE m18 expanded to transient immunodominance between weeks 3 and 4, concomitant with the above discussed sharp increase in the overall proportion of CD8^+^ T cells in the lung infiltrates (recall Figure 1B). Accordingly, concomitant with the subsequent decline in the overall proportion, m18-specific CD8^+^ T cells vanished and never reappeared.

Honestly, we do not really know why clones of a certain epitope specificity flash and become extinct. General availability of growth factors for clonal expansion, such as interleukin-2, is unlikely an explanation, as other specificities did not profit at the same time. We also do not see any rationale for a transiently preferential presentation of the m18 epitope during productive infection of the lungs. We must rather propose an intrinsic clonal property that may even be independent of the epitope. Although an explanation is missing, we must note the here observed fact of a “coming and going” of epitope-specific antiviral responses in the phase of immune reconstitution after HCT.

Interestingly, in studies on primary hCMV infection of otherwise healthy immunocompetent individuals, dynamics by selection of clonotypes has also been identified as a characteristic of the CD8^+^ T-cell response [reviewed in Ref. (52)].

### Deletion of IDEs: Rationale, Predictions, and Experimental Verification

**Figure 3:**
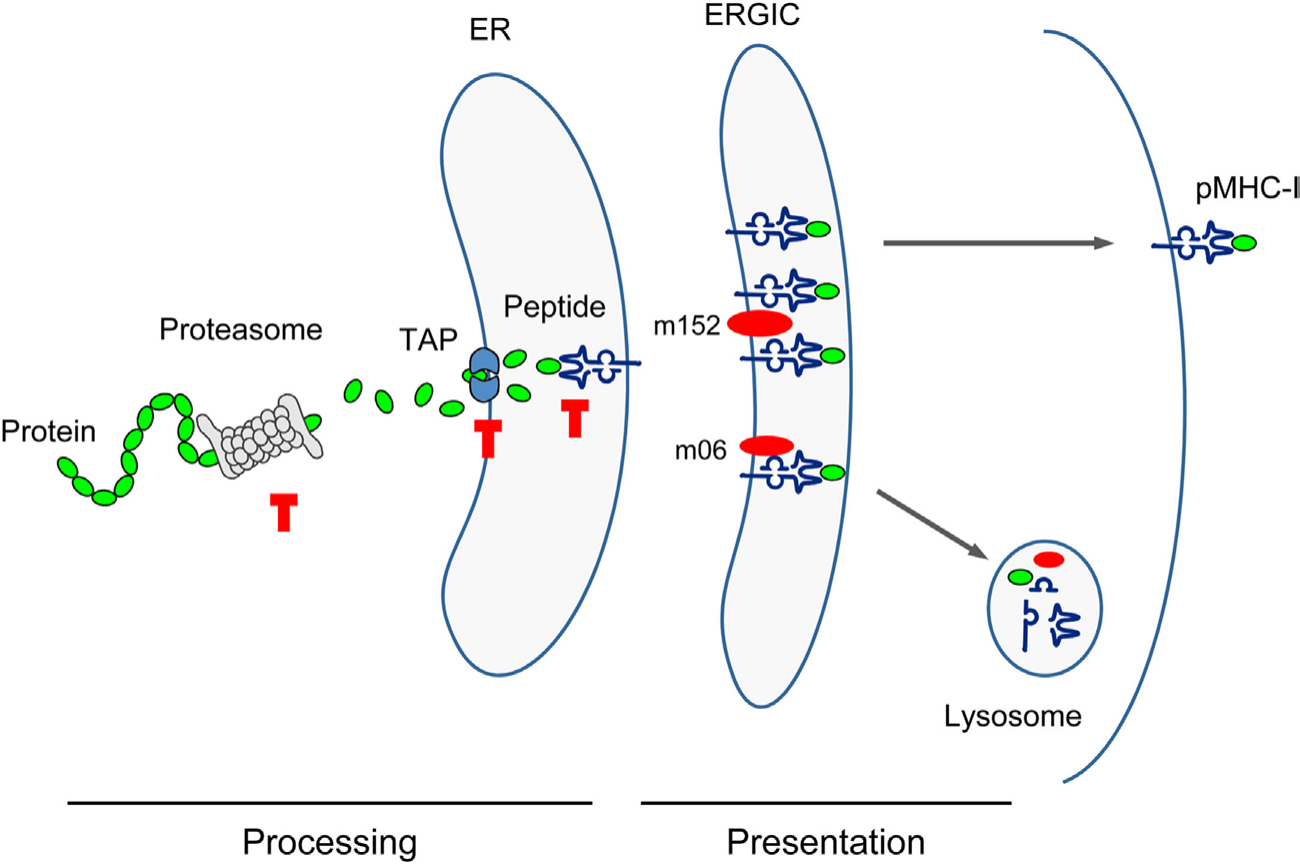
**Sketch of antigen processing and presentation**. Buffer stop symbols indicate steps at which substitution of the antigenic peptide’s C-terminal amino acid with Ala (X9Ala) could block peptide processing. TAP, transporter associated with antigen processing/presentation; ER, endoplasmic reticulum; ERGIC, ER-Golgi intermediate compartment. Red oval symbols indicate immune evasion proteins interfering with vesicular flow trafficking of pMHC-I complexes.

**Figure 4:**
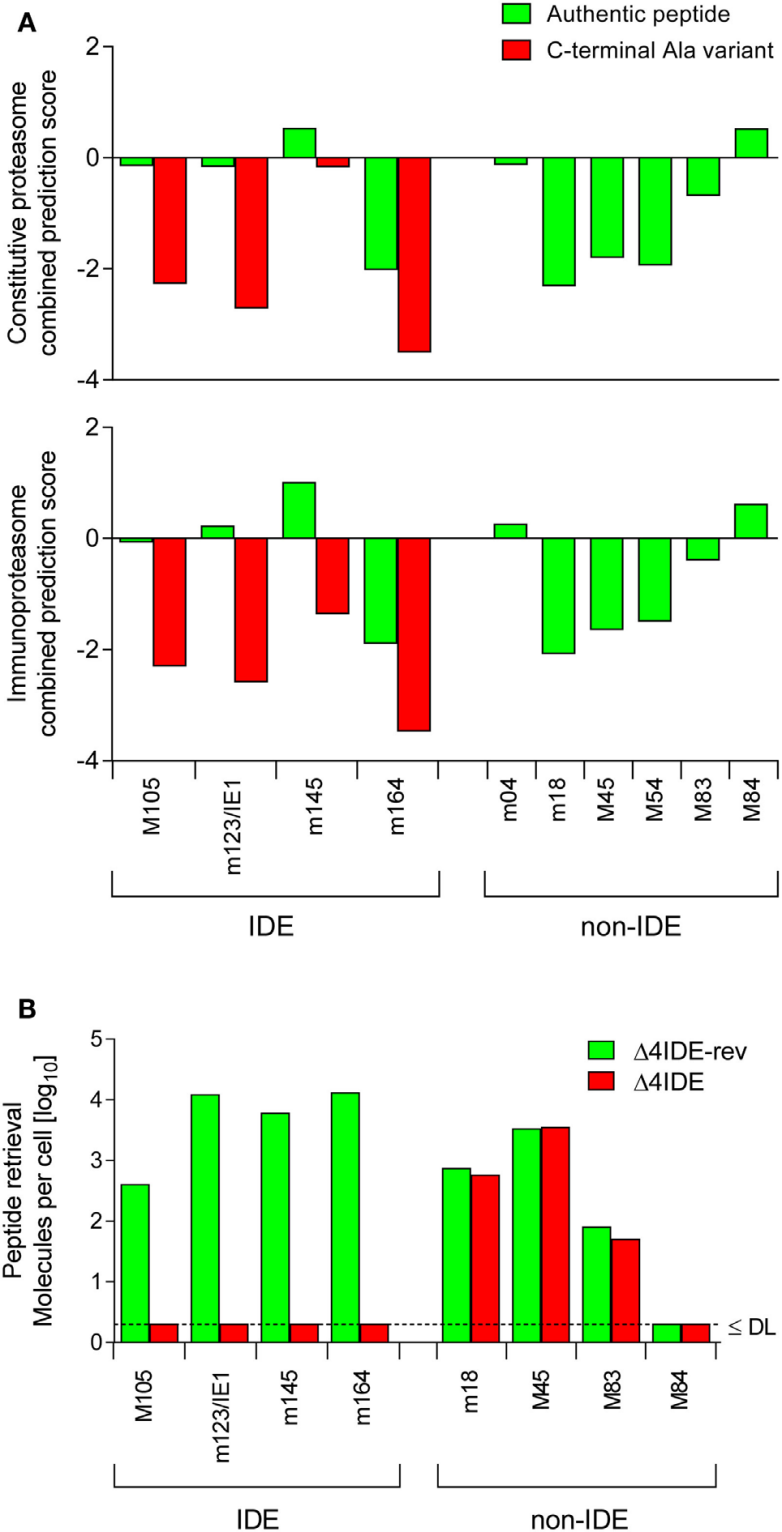
**Predicted and experimental efficacies of peptide processing**. **(A)** Graphs of combined prediction scores. Processing predictions (proteasomal cleavage/TAP transport/MHC class-I combined predictor) were made using the IEDB analysis resource. Shown are scores for immunodominant (IDE) and non-immunodominant (non-IDE) epitopes. Authentic peptides (green bars). C-terminal amino acid substitutions with Ala (red bars) reduce the score. **(B)** Yield of antigenic peptides from MEF infected with mutant virus mCMV-Δ4IDE (red bars), lacking IDEs, and the corresponding revertant virus mCMV-Δ4IDE-rev expressing IDEs (green bars). DL, detection limit.

Our group has already pursued the strategy to alter immunogenicity and antigenicity of mCMV by functional deletion of epitopes using site-directed BAC-mutagenesis to introduce a point mutation that replaces the MHC-I anchoring C-terminal residue of an antigenic peptide with alanine, X9Ala [(25, 43, 56, 57, 90); for a review of the concept, see Ref. (91)]. Unlike deletion of the complete peptide-coding sequence, this strategy promises to be “minimally invasive” in that the intended immunological phenotype can be achieved with minimal alteration of the corresponding protein and, thus, with the lowest risk of a loss of its physiological function. In previous work, cited above, we have emphasized the impact of this mutation on anchoring the peptide in the hydrophobic pocket of the presenting MHC-I molecule to yield a pMHC-I complex (92–94). This is still true as an utmost important parameter, as shown by predicted loss of binding affinity to MHC-I, indicated by an increase in the IC50 value (Table 2). However, this mutation can in addition already reduce peptide generation by inefficient proteasomal cleavage as well as inefficient peptide transport into the ER via the TAP prior to peptide loading onto nascent MHC-I molecules (for a sketch, see Figure 3; for prediction scores, see Table 2). The critical bottlenecks in the pathway may vary between different peptides (boxed in Table 2). Combined processing scores for all known antigenic peptides in the *H-2^*d*^* haplotype, IDEs and non-IDEs (Table 1), which include all steps from proteasomal processing to peptide loading (Figure 3), are graphically summarized in Figure 4A. This illustrates the predicted effect of the C-terminal mutations I215A, L176A, L459A, and I158A in the IDEs M105, m123/IE1, m145, and m164, respectively. In this regard, there are no fundamental differences predicted between constitutive proteasome and immunoproteasome. As a side aspect, the predictions also show that a poor processing score does not preclude a peptide to be antigenic. This is probably due to favorable parameters operative at steps before entering the proteasome, such as high protein amount, low stability, and efficient ubiquitination for proteasomal import and degradation. In theory, the introduced mutation to Ala might lead to misfolding of the protein and decreased stability, a factor rather expected to enhance proteasomal degradation (95).

**Table 2:** **Predicted influence of X9Ala replacements on steps in peptide processing**.^a^

ORF	Antigenic peptide	Score						
		Proteasome		TAP	MHC-I	Total		MHC-I IC50 [nM]
		const	immuno			const	immuno	
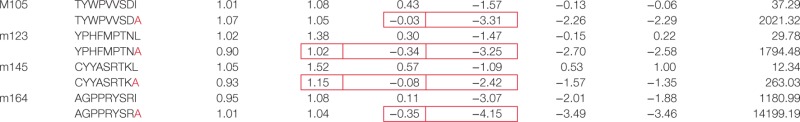								

*^a^Processing predictions (proteasomal cleavage/TAP/MHC class-I combined predictor) were made using the IEDB analysis resource. Steps more critically affected by X9Ala replacements are highlighted by red boxes*.

Cell surface presentation of pMHC-I complexes for recognition by the TCR of CD8^+^ T cells is inhibited by mCMV immune evasion proteins m152, retaining complexes in a cis-Golgi/ER-Golgi intermediate compartment (ERGIC), and m06, rerouting the complexes to late endosomes/lysosomes for degradation [reviewed in Ref. (31, 96)]. This interference with the vesicular trafficking of pMHC-I complexes, however, is not thought to be affected by the C-terminal Ala.

The predictions were experimentally verified by isolating the corresponding peptides from lysates of MEF infected with mutant virus mCMV-Δ4IDE (57) and the corresponding revertant virus mCMV-Δ4IDE-rev (70) (Figure 4B). IDE-representing peptides could no longer be isolated when cells were infected with the mutant virus, whereas infection with the revertant virus yielded high amounts of peptide per cell (up to 10^4^ m123/IE1 and m164 molecules each). It should be noted that peptide yield reflects the number of the corresponding pMHC-I complexes, as peptides that fail to bind to MHC-I molecules are degraded. It must be considered that peptide yield is proportional to processing efficacy, but is not identical to the amount of processed peptides, because immune evasion molecule m152 increases the yield (76) by arresting the pMHC-I complexes in the ERGIC, while m06 is supposed to reduce the yield by lysosomal degradation (Figure 3). To our knowledge, the net outcome of these opposite effects of the two immune evasion proteins has never been quantitated. So, with this question mark in mind, we operationally take peptide yield as an approximation for processing.

Importantly, deletion of the four IDEs had no influence on the yield of peptides representing a panel of non-IDEs, namely m18, M45, M83, and M84 (Figure 4B). This means that there is no notable competition between IDEs and non-IDEs at the levels of proteasome and TAP. Furthermore, as each non-IDE has an IDE counterpart using the same presenting MHC-I molecule (Table 1), there appears to be also no competition at the level of MHC-I binding. Finally, with the exception of M84, the amounts of non-IDE peptides per cell are not generally much lower than that of the IDE peptides, in particular when we consider IDE peptide M105.

**Figure 5:**
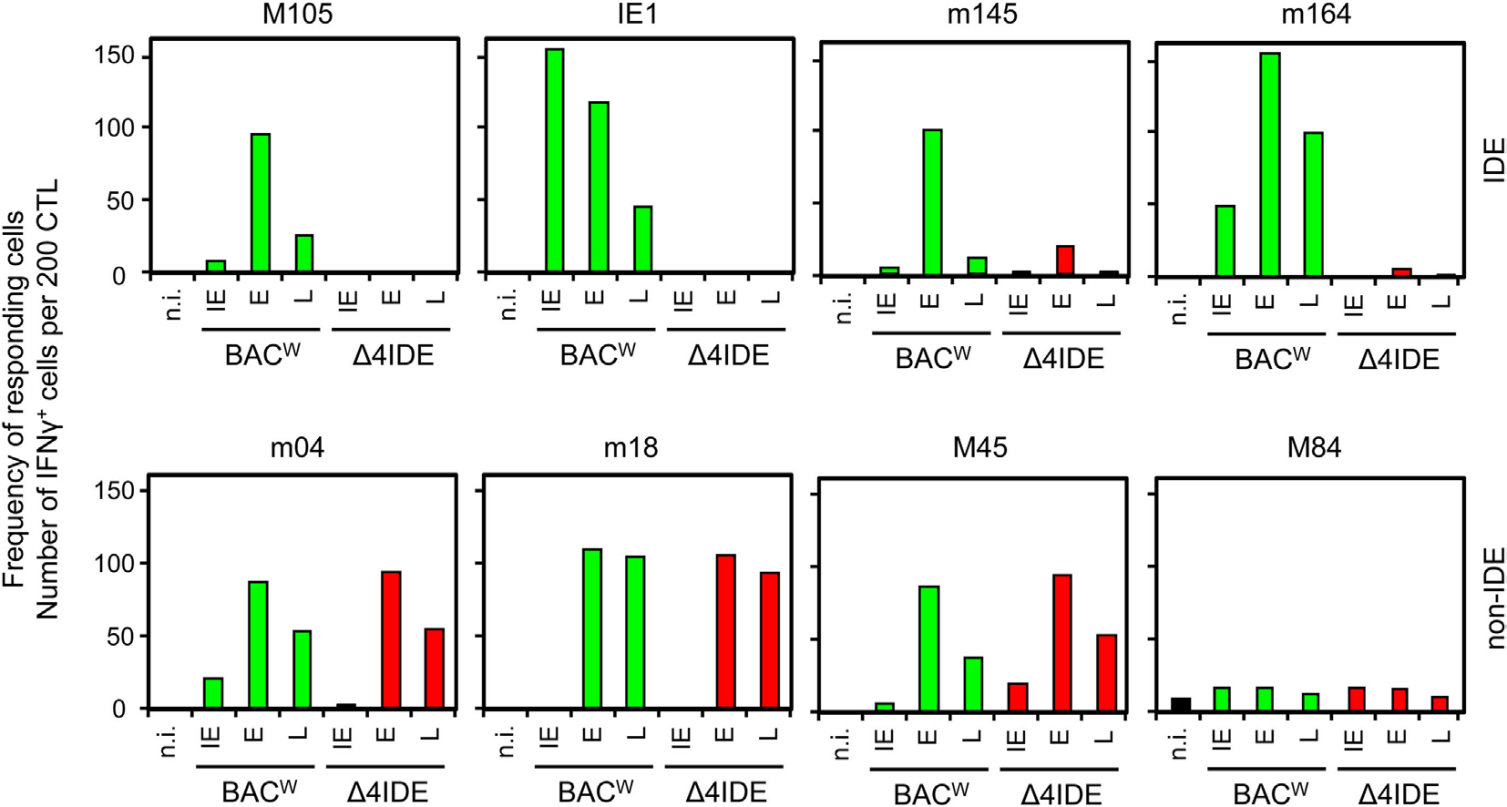
**No impact of IDE deletion on antigenic peptide presentation**. MEF were infected with BAC-derived parental virus mCMV-BAC^W^, expressing IDEs (green bars), or mutant virus mCMV-Δ4IDE (red bars), lacking IDEs. Infected MEF presenting antigenic peptides derived from the three kinetic phases of viral gene expression, “immediate early” (IE), “early” (E), and “late” (L), were used as stimulator cells in an ELISpot assay. Cells of epitope-specific CTLL were used as responder cells. Bars represent frequencies of CTL responding to the stimulation with secretion of IFN-γ.

Functional deletion of the IDEs was confirmed by testing the cell surface presentation of IDE–pMHC-I complexes on cells infected with IDE-expressing BAC-derived virus mCMV-BAC^W^ (69) or mutant virus mCMV-Δ4IDE via recognition by the cognate CTLL as determined by IFN-γ secretion upon stimulation in an ELISpot assay (Figure 5, upper panels). To account for different kinetic classes of viral protein expression, stimulator cells in the assay were metabolically arrested in the “IE” phase, or in the “E” phase, or were allowed to proceed to the “late” (L) phase. As a remark, IE phase expression of the antigenic peptide assigned to the E Phase protein m164 has recently been shown to result from a transcript starting within an upstream IE gene (89). With the exception of a minor residual recognition of the Ala-variant L459A of the m145 E-phase peptide, which is paralleled by a still high constitutive proteasome combined processing score (Figure 4A, upper panel), the recognition of IDEs was abolished by the C-terminal replacements with Ala.

Finally, with the corresponding non-IDE-specific CTLL, we compared presentation of non-IDE pMHC-I complexes in presence or absence of IDEs after infection with viruses mCMV-BAC^W^ and mCMV-Δ4IDE, respectively (Figure 5, lower panel). At a glance, presence or absence of IDEs had no notable effect on the presentation of four tested non-IDEs. Specifically, presentation of non-IDEs did not profit from the absence of IDEs. This leaves us with the conclusion that the phenomenon of “immunodominance” is not regulated at the levels of antigen processing or presentation.

### Absence of IDEs Does Not Negatively Influence Control of Infection after HCT

**Figure 6:**
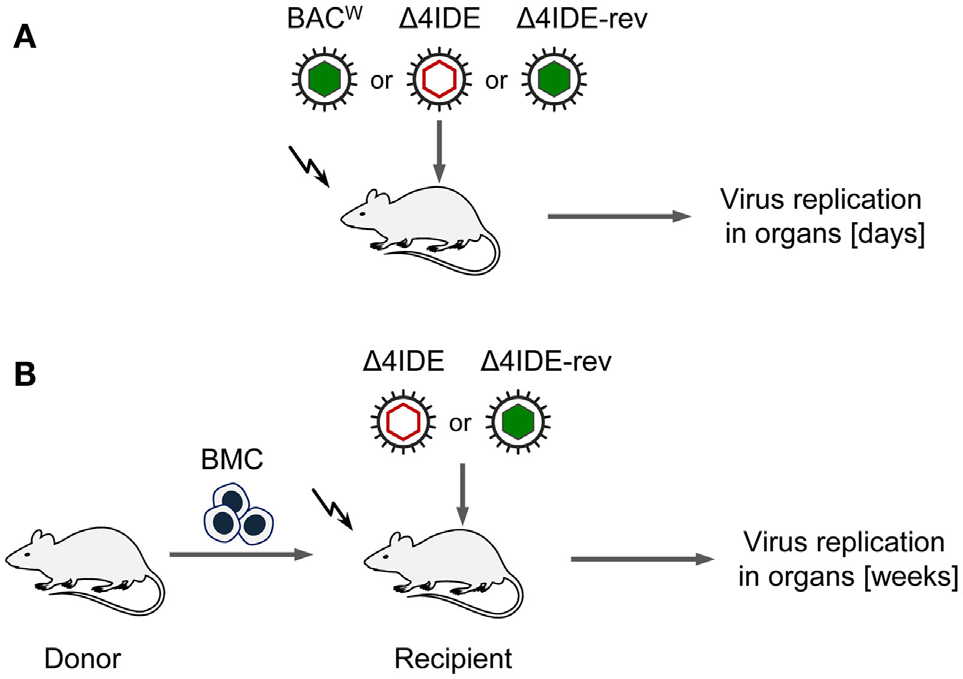
**Sketch of the experimental regimens**. **(A)** Infection of BALB/c mice (destined as recipients for HCT), immunocompromised by hematoablative total-body γ-irradiation (flash symbol) but with no HCT being performed. Filled and empty capsid virus pictograms indicate presence and absence of IDEs, respectively. **(B)** HCT performed by intravenous transfer of bone marrow cells (BMC).

We initiated this study with the question if absence of an IDE-specific CD8^+^ T-cell reconstitution would prevent control of infection after HCT as seen previously after pan-CD8^+^ T-cell depletion [(44, 45), see also the accompanying Review article in this issue of *Frontiers in Immunology*]. To approach this question, we compared the infection of immunocompromised recipients with viruses expressing or not expressing IDEs first in the absence of hematopoietic reconstitution and then in the presence of hematopoietic reconstitution after HCT (for a sketch of the experimental models, see Figures 6A,B, respectively).

**Figure 7:**
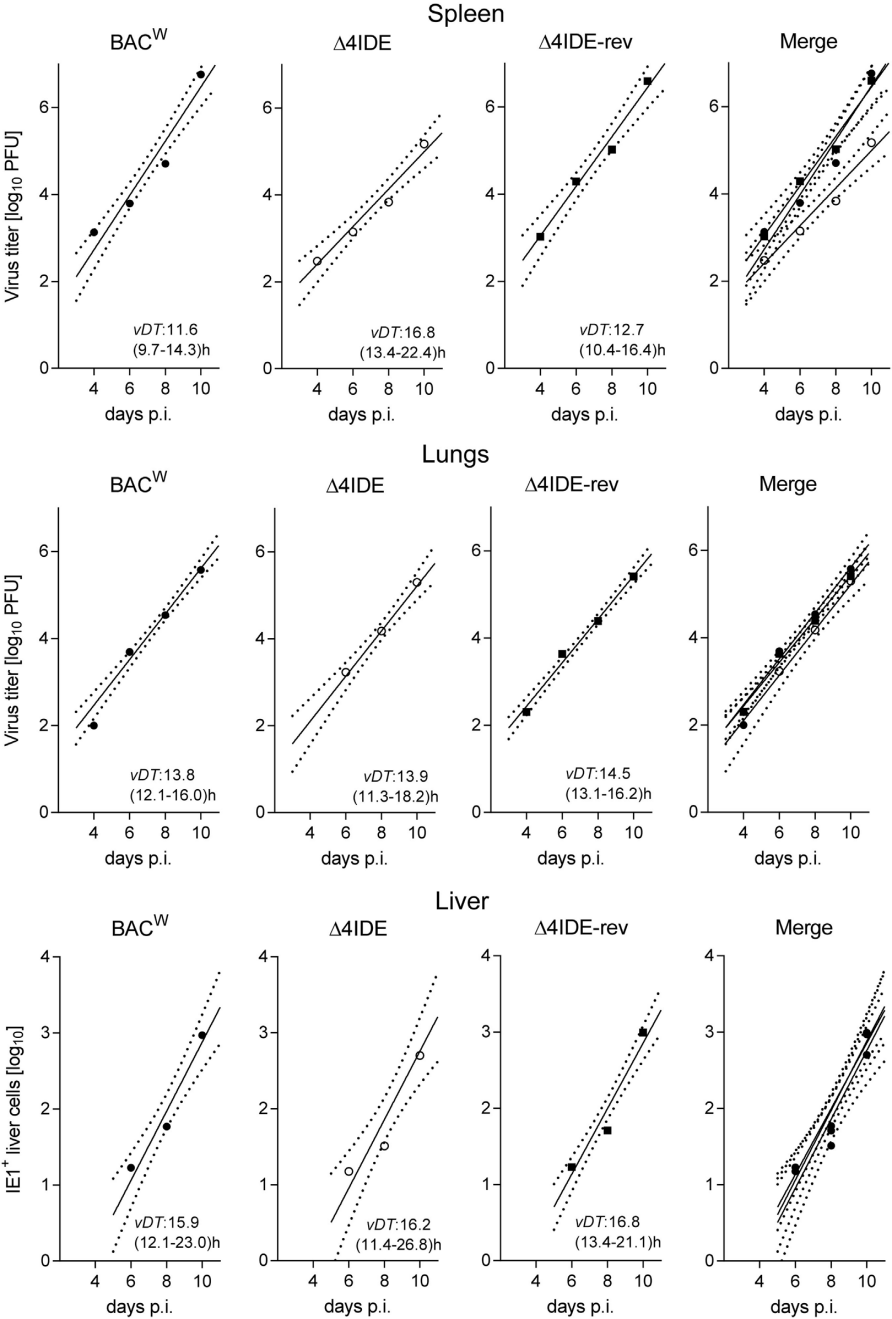
**Viral replicative fitness in organs of immunocompromised mice**. Following regimen “A” of Figure 6, log-linear growth curves in the spleen (top), lungs (center), and liver (bottom) were determined over a period of 10 days for the three viruses indicated. For graphical clarity, symbols depict only the median values of data from three mice per time point measured, though regression lines were calculated by including data from all individual mice and all assay times. Dotted lines demarcate the 95% confidence areas of the corresponding regression lines. Virus attenuation is indicated by an increased value of the viral doubling time, *vDT*, and thus by a reduced slope of the regression line. The 95% confidence intervals for *vDT* are given in parentheses. p.i., post-infection. Virus titers in spleen and lungs refer to the whole organ, the numbers of IHC-detected, infected (IE1^+^) liver cells (mostly hepatocytes) refer to representative 10 mm^2^ areas of liver tissue sections.

Testing infection after hematoablative treatment, with no HCT being performed, is important for evaluating viral replicative fitness *in vivo* in order to detect potential attenuation of mutant virus for reasons unrelated to reconstitution of immune control. Although the above described C-terminal replacements with Ala in the IDE peptide sequences are the minimal procedure required to achieve the intended immunological loss-of-presentation phenotype, one can never exclude an effect on protein folding and function. As explained in detail in recent reports (97, 98), virus spreads and replicates exponentially over time in organs of an immunodepleted host, which leads to log-linear growth regression lines with the vDT as the growth constant that reveals replicative fitness. Unlike growth curves in cell culture, this *in vivo* approach has the advantage to incorporate all cell types that constitute the tissues of tested organs in the natural microanatomical context. This experimental protocol (Figure 6A) revealed a minor increase in vDT (slower replication), and, thus, a slight attenuation, of mutant virus mCMV-Δ4IDE in the spleen compared to mCMV-BAC^W^ (Figure 7, upper panels). This attenuation was clearly related to the mutations since the growth-deficiency phenotype of mCMV-Δ4IDE was reversed to normal growth in the IDE-expressing revertant virus mCMV-Δ4IDE-rev. Although we could map this attenuation to the M105-Ala variant I215A (data not shown), impaired helicase-primase function of protein M105 (99) is unlikely the reason for growth attenuation in the spleen, as no attenuation of mCMV-Δ4IDE was seen in lungs and liver of the same mice (Figure 7, center and lower panels, respectively). We did not further investigate the reason for the spleen-selectivity of this attenuation, but it likely relates to infected cell type(s) present in the spleen but not in lungs and liver, or to a higher residual innate immunity in the spleen. It should be noted that in absence of HCT, immunodepleted mice die of multiple-organ CMV disease from day 10 onward (35).

**Figure 8:**
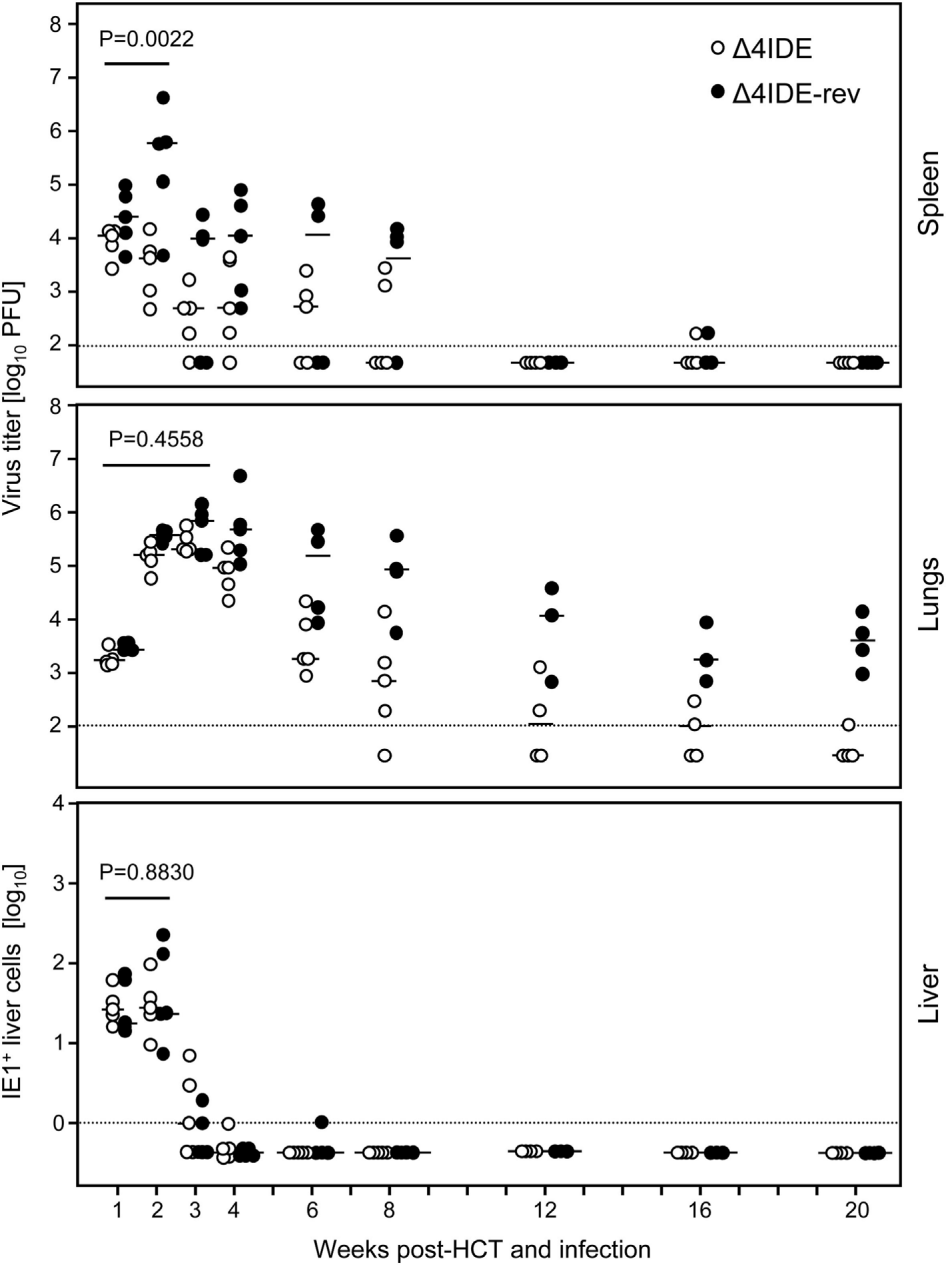
**Influence of IDEs on the course of infection in HCT recipients**. Following regimen “B” of Figure 6, virus replication in HCT recipients infected with mCMV-Δ4IDE, lacking IDEs (open circles), or mCMV-Δ4IDE-rev, expressing IDEs (filled circles), was quantitated over a period of 20 weeks in the spleen (top), lungs (center), and liver (bottom) during hematopoietic reconstitution. Symbols represent data from individual mice, dashes represent the median values. Virus titers in spleen and lungs refer to the whole organ, the numbers of IHC-detected, infected (IE1^+^) liver cells (mostly hepatocytes) refer to representative 10-mm^2^ areas of liver tissue sections. *P*-values compare data for mutant virus (open circles) and revertant virus (filled circles) cumulated for the time points that precede the reconstitution of CD8^+^ T cells.

With these results in mind, we extended the model by performing HCT, that is by transfer of syngeneic BMCs to the recipients after hematoablative conditioning and followed by intraplantar infection (Figure 6B). The reconstitution led to survival of the recipients with control of both viruses, mCMV-Δ4IDE and mCMV-Δ4IDE-rev, in spleen, lungs, and liver (Figure 8). In the spleen, the IDE-deletion mutant replicated to lower levels already in the first 2 weeks after HCT (Figure 8, upper panel, *P* = 0.0022), before reconstitution of CD8^+^ T cells becomes detectable (data not shown). Thus, in accordance with the slower replication in the spleen of immunodepleted, unreconstituted mice (recall Figure 7, upper panel), subsequent immunological control of mCMV-Δ4IDE in the spleen is obscured by the site-specific attenuation of this mutant. In the lungs, however, this caveat does not apply, as mutant and revertant virus replicated there with no difference until week 3 (Figure 8, center panel; *P* = 0.4558), the time when reconstituted CD8^+^ T cells first appear in the lungs (recall Figures 1 and 2). Surprisingly, at the peak of CD8^+^ T-cell infiltration at 4 weeks and at all later times, the mutant virus was controlled more efficiently than the revertant virus and became latent between 16 and 20 weeks when the revertant virus was still in low-level persistent replication. Finally, in the liver, both viruses replicated equally at all times, with latency being established for both viruses from week 4 onward.

**Figure 9:**
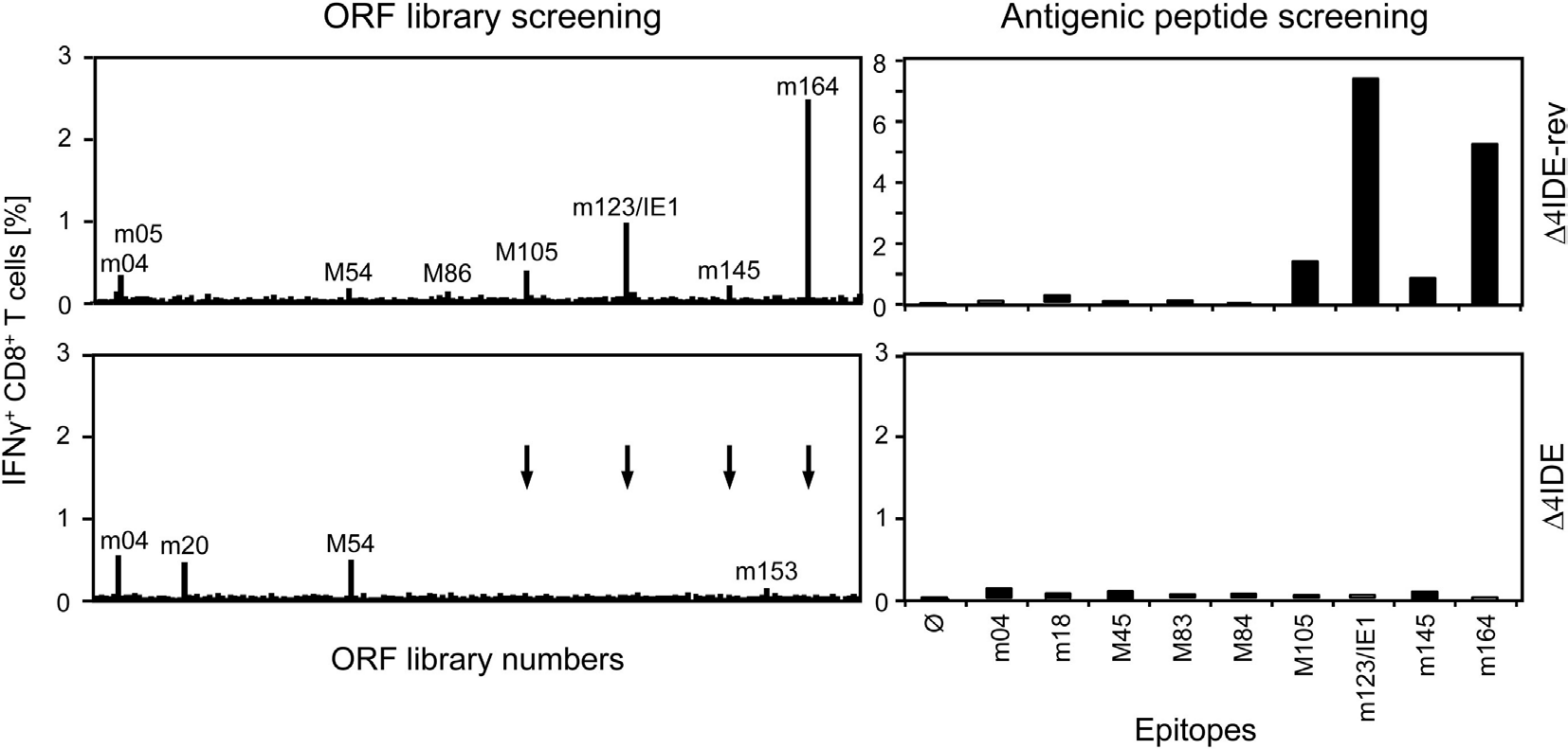
**Deletion of IDEs is not associated with global alteration in epitope-recognition patterns**. At 6 months after HCT and infection with mCMV-Δ4IDE-rev, expressing IDEs (upper panels), or mCMV-Δ4IDE, lacking IDEs (lower panels), epitope-specific reconstitution of CD8^+^ T cells in the spleen was monitored and quantitated by cytofluorometric intracellular IFN-γ assays of cells responding to sensitization. (Left panels), mCMV genome-wide ORF library screening. (Right panels), screening with synthetic peptides corresponding to known epitopes. Arrows point to ORF library positions of epitopes deleted in mutant virus mCMV-Δ4IDE.

We determined the status of epitope-specific CD8^+^ T-cell reconstitution in the latently infected spleens by stimulation with a genome-wide open reading frame (ORF) library of transfected cells (Figure 9, left panels) and by stimulation with saturating doses of synthetic peptides representing the known epitopes (Figure 9, right panels), in both cases measuring the frequency of stimulated CD8^+^ T cells by intracellular IFN-γ. In the case of infection with mCMV-Δ4IDE-rev the two epitopes long known as epitopes that induce MI, namely m123/IE1 and m164 (see above), dominated the memory pool, but memory cells specific for the more recently defined IDEs M105 and m145 were also detectable in both assays. In addition, minor responses were detected by the ORF library approach to ORFs m05, M54, and M86. After infection with mutant virus mCMV-Δ4IDE, IDE-specific memory cells were missing as expected. Interestingly, although the ORF library revealed minor reactivity to ORFs m04, m20, M54, and m153, none of the known non-IDE epitopes appeared to notably profit from the absence of IDEs. Note that, at the time of the experiment, the M54 peptide was not yet known and has in the meantime been described as an epitope that indeed profits from the absence of IDEs (70), in accordance with its enhanced appearance in the ORF library assay.

In conclusion, the results were clear in that IDEs were not essential for control of infection after HCT and were not even of advantage, as at least pulmonary infection was controlled even somewhat better in the absence of IDEs.

### CD8^+^ T Cells Specific for Non-IDEs Form Protective Nodular Inflammatory Foci

The data thus far gave the unequivocal message that absence of IDE-specific CD8^+^ T cells during reconstitution after HCT differs fundamentally from a pan-CD8^+^ T-cell depletion in that the recipients still control the infection and survive, whereas the pan-depletion led to lethal multiple-organ CMV disease in 100% of the HCT recipients (see the accompanying Review article in this issue of *Frontiers in Immunology*). Our proposed explanation is that CD8^+^ T cells specific for many non-IDEs, most of which may have an individual frequency below assay detection limit, collectively mount an efficient antiviral response. As we have shown previously, antiviral protection has a microanatomical correlate in the formation of NIF in which CD8^+^ T cells gather around infected tissue cells for confining the infection by preventing intra-tissue spread (45, 100, 101). Importantly, the formation of NIF is epitope specific in that infected cells must present the cognate epitope (43, 90). We, therefore, addressed the question if non-IDE-specific CD8^+^ T cells can form NIF in mice infected with mCMV-Δ4IDE, which would indicate that the non-IDEs are indeed presented *in situ* by infected tissue cells and recognized in an epitope-specific manner by cognate CD8^+^ T cells present in a cell population devoid of IDE-specific cells.

**Figure 10:**
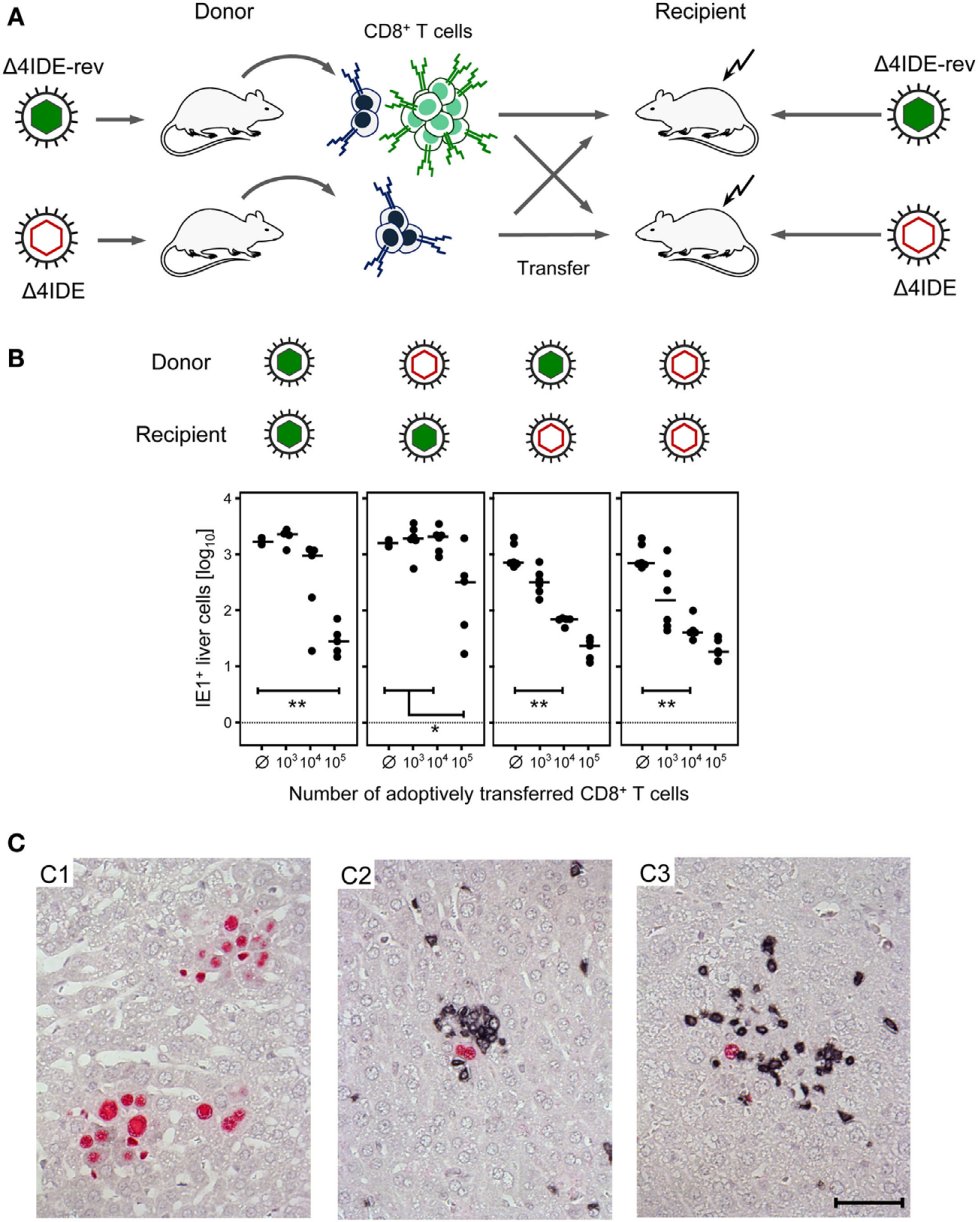
**Non-IDE-specific recognition of infected host tissue cells identified by the formation of protective nodular inflammatory foci (NIF)**. **(A)** Sketch of the experimental strategy of criss-cross adoptive transfer of spleen-derived memory CD8^+^ T cells primed in presence or absence of IDEs into recipient mice presenting or not presenting IDEs on their tissue cells after infection with the viruses mCMV-Δ4IDE-rev and mCMV-Δ4IDE, respectively. **(B)** Control of liver infection in the four possible transfer combinations. The numbers of IHC-detected, infected (IE1^+^) liver cells (mostly hepatocytes) were determined on day 11 and refer to representative 10-mm^2^ areas of liver tissue sections. Filled circles represent individual mice, with the respective median values marked by a dash. Ø, no cells transferred. Statistical significance of differences is indicated comparing groups of interest. **(C)** Two-color IHC with red staining of intranuclear viral IE1 protein, identifying infected cells, and black staining of CD3ϵ, identifying tissue-infiltrating T cells. (C1) Foci of infection with mCMV-Δ4IDE-rev formed by many infected (red) cells in the group with no CD8^+^ T-cell transfer. Images for infection with mCMV-Δ4IDE are alike. (C2) NIF formed by infiltrating CD8^+^ T cells (black) in the transfer combination IDE:IDE, indicating confinement of the infection to single infected cells (red). (C3) NIF formed by infiltrating non-IDE-specific CD8^+^ T cells (black) in the transfer combination non-IDE:non-IDE, likewise confining the infection to single infected cells (red). The bar marker applies to all images and represents 50 μm.

We approached this question in the previously described criss-cross adoptive CD8^+^ T-cell transfer/immunotherapy model (56, 57) by using mutant or revertant virus for donor mouse priming as well as for the infection of recipients immunocompromised by hematoablative treatment (Figure 10A). In essence, infection of the liver was controlled in all four donor–recipient combinations. Notably, the control was somewhat more efficient in mCMV-Δ4IDE-infected recipients, regardless of the donor IDE equipment, and least efficient when IDE-specific cells were missing in the donor cell population transferred into recipients presenting IDEs. Most importantly, liver infection was efficiently controlled also by the CD8^+^ T-cell population that is devoid of IDE-specificity and even upon transfer into recipients not presenting IDEs on the infected cells (Figure 10B). In absence of CD8^+^ T-cell transfer, infection was not controlled and NIF were not formed (Figure 10C, image C1), which excludes protective NIF formation by residual other potentially CMV-protective cell types of innate or adaptive immunity, including cells also expressing CD3ϵ detected in the two-color IHC, such as CD4^+^ T cells, γ/δ T cells, and NKT cells. Importantly, protective NIF were formed by the tissue-infiltrating CD8^+^ T cells in all four cell transfer groups, as shown representatively for the IDE:IDE combination as the positive reference scenario (Figure 10C, image C2) and for the non-IDE:non-IDE combination as the group under investigation (Figure 10C, image C3).

In conclusion, non-IDE-specific CD8^+^ T cells can recognize their cognate non-IDE epitopes presented on infected host tissue cells for the formation of NIF that confine and eventually terminate tissue infection.

## Discussion

The mouse model of CMV infection [reviewed in Ref. (34)] has proven its validity for approaching medical questions that cannot be addressed in patients and that are logistically too demanding to be readily approached in non-human primate models [for reviews, see Ref. (6, 102)]. Considering advantages and limitations of any model, the mouse model, to the very least, can make predictions for specific evaluation in lower sample numbers of non-human primates or in observational clinical studies (for a more detailed discussion, see the accompanying Review article in this issue of *Frontiers in Immunology*).

It is a long-established clinical observation that reconstitution of an antiviral CD8^+^ T-cell response correlates with control of reactivated hCMV infection after HCT (29, 30). It is also known that a limited but individually distinct set of viral epitopes, the IDEs, dominates the antiviral response in quantitative terms in any individual person based on her/his individual immunogenetic makeup and virus strains harbored in latency (46). This has led to the logical opinion that monitoring of immunoreconstitution after HCT, vaccine development, and cytoimmunotherapy should aim for IDE-specific CD8^+^ T cells. Focus on IDEs is further supported by the relative ease to detect and quantitate these responses and to sort a sufficient number of epitope-specific CD8^+^ T cells for adoptive immunotherapy, an approach principally successful in human trials (40, 41) and in the mouse model (36). Although IDE-specific CD8^+^ T cells can undoubtedly be protective and are, thus, not a “wrong” choice, the question remained if antiviral protection depends on IDEs, and if IDEs are necessarily the “best” choice.

Our own previous work on cytoimmunotherapy in the mouse model, specifically adoptive transfer of non-IDE-specific CTLL or polyclonal memory CD8^+^ T cells devoid of IDE-specificities [(57), and reviewed in Ref. (32–34)], corroborated by studies of D.H. Spector´s group on genetic vaccination in the immunocompetent mouse challenge model (55), indicated an at least equal or even better antiviral protection exerted by CD8^+^ T cells recognizing non-IDEs. This finding is in accordance with other systems reviewed recently (103). In CMV infections, prediction of the protection-inducing capacity of an epitope is further complicated by an additional parameter, namely by the action of immune evasion molecules that interfere with cell surface presentation of pMHC-I complexes. This has led to the notion that antigens and “immunoevasins” are opponents in CMV immune surveillance (31). As a most prominent example for a “non-protective” IDE, a CTLL specific for the mCMV ORF M45-derived D^b^-presented peptide HGIRNASFI (104) fails to protect immunocompromised C57BL/6 mice, unless immune evasion molecule m152 is deleted to allow cell surface presentation of pM45-D^b^ complexes (105). Interestingly, the inhibitory effect of immune evasion molecules is linked to peptide processing efficacy in infected cells in that inhibition is complete for the poorly processed M45 peptide (76), but becomes leaky when peptide processing is enhanced by IFN-γ to yield higher numbers of pM45-D^b^ complexes saturating the inhibitory capacity of immune evasion molecules (106). More recently, the K^d^-presented ORF M105 epitope (Table 1) has been identified as a second example of a non-protective IDE that turns into being protective when immune evasion molecules are deleted (33).

Here, we have expanded our previous work in the murine model by addressing the question if IDE-specificity of CD8^+^ T cells is essential for controlling CMV infection in the specific context of immunoreconstitution after HCT when T-cell lymphopoiesis, thymic selection, and priming take place under conditions imposed by the infection, including an altered cytokine-chemokine milieu. This is a situation that has a clinical correlate and that differs essentially from adoptive transfer models that are based on already differentiated memory or effector cells. Studying the role for IDEs under conditions of HCT is particularly important in the light of previous findings that CD8^+^ T cells are dispensable for CMV control in selectively depleted but otherwise immunocompetent mice (107, 108), whereas depletion of CD8^+^ T cells during immunoreconstitution in infected HCT recipients is inevitably lethal (44, 45). We, thus, began our study with the working hypothesis that a failure to reconstitute a quantitatively dominant IDE-specific CD8^+^ T-cell response might be as lethal as pan-CD8^+^ T-cell depletion or might end up in persistent productive infection or at least in a delayed clearance of productive infection. The data are unequivocal in demonstrating that IDE-specific immunoreconstitution is neither essential nor of additional benefit.

Epitope-specific immunomonitoring of antiviral CD8^+^ T cells reconstituted following syngeneic HCT revealed that the epitope-recognition patterns vary between HCTs performed under nominally identical conditions, and are highly dynamic over time within each HCT, with the only constant feature that at late times the initially broader response focuses on the MI-inducing epitopes. Strong clonal expansions and high dynamics have been described also for the T-cell response to hCMV [reviewed in Ref. (52)]. Interestingly, in HCT #3 (Figure 2), CD8^+^ T cells specific for the non-IDE epitope m18 expanded to high numbers between weeks 3 and 4, so that one could have classified it as an IDE. However, this expansion was transient and the m18 response was completely lost thereafter. It appears as if certain CD8^+^ T-cell clones stochastically can gain a temporary growth advantage, independent of their classification as being IDE- or non-IDE specific, a classification that is usually based on the specificity hierarchy in immunocompetent mice. It is worthwhile considering that an unintended encounter with unrelated antigens during the long observation period can shape the memory pool specific for the pathogen under investigation, a parameter of epitope hierarchy dynamics and response plasticity experimentally documented by the group of Welsh (109, 110). Such an influence varies unpredictably between different HCTs, and obviously, encounters with unrelated antigens are unavoidably the rule in the medical reality of patients. The finding that the response to MI-inducing epitopes prevails on the long run is explained by repetitive intrinsic episodes of antigenic peptide presentation and memory T-cell restimulation based on sporadic viral gene expression events during latency, which can be driven by inflammatory cytokine signaling during pathogen encounters [(111); reviewed in Ref. (27, 28)].

Genetic deletion of the four prominent IDEs of mCMV presented in the *H-2^*d*^* haplotype was here found to have no significant impact on the processing and presentation of non-IDEs in infected cells, which excludes competition between IDEs and non-IDEs at any step of this pathway (Figure 3) as a reason for immunodominance, a conclusion that is in accordance with current opinion [reviewed in Ref. (103)]. Notably, although immunodominance could be defined at the level of TCR-pMHC-I interaction driving T-cell expansion, we did not observe notable and reproducible expansion of CD8^+^ T cells specific for defined non-IDEs in the absence of IDEs upon infection of mice with mutant virus mCMV-Δ4IDE during either acute or latent infection in the spleen [(57) and this report, respectively]. Moreover, genome-wide ORF library screenings did not indicate a broad emergence of new specificities in the spleen during either acute or latent infection [(57) and this report, respectively], with the notable exception of M54 in the lungs during acute infection after HCT (70). Nonetheless, absence of IDEs did not impair the control of infection after HCT (Figure 8). A hint to an explanation is given by previous work on the deletion of the two MI-inducing IDEs, m123/IE1 and m164 (56), showing that epitope hierarchies change when related to functional avidity, and that high quantity defining “immunodominance” is based primarily on low-avidity cells detected with high concentrations of exogenously loaded synthetic peptides. As presentation of naturally processed antigenic peptides in host tissue cells is a limiting factor, and as high-avidity is a predictor for protective activity *in vivo* (33, 112), low-avidity responses to IDEs likely contribute little to protection. Work in progress indicates a gain of avidity in the pool of non-IDE-specific CD8^+^ T cells primed in the absence of IDEs (Rafaela Holtappels, preliminary data not shown), which would offer an explanation for the more efficient control of mutant virus mCMV-Δ4IDE in the lungs (Figure 8).

The critical question remained, however, if non-IDE-specific CD8^+^ T cells protect by recognition of non-IDEs presented on infected host tissue cells or if deletion of IDEs might have called up alternative mechanisms of immune control. Antiviral protection *in vivo* has a microanatomical correlate in the formation of NIF, clusters of focally infiltrating CD8^+^ T cells that attack infected tissue cells and thereby confine and eventually clear productive infection (referenced above). Importantly, NIF formation requires presentation of the cognate epitope (90). Here, our presented finding that protective NIF are formed by non-IDE-specific CD8^+^ T cells in liver tissue infected by mCMV-Δ4IDE verifies protection by recognition of presented non-IDEs.

In conclusion, our study revealed that IDEs are not essential for control of CMV infection in the context of HCT. Importantly, this implies that antigenicity/immunogenicity-loss mutations in IDEs will not likely lead to immune escape of the virus. For adoptive immunotherapy, this predicts a robustness of the therapy toward antigenically relevant proteomic differences in the hCMV strains harbored in latently infected HCT patients.

## Ethics Statement

Animal experiments were approved by the ethics committee of the “Landesuntersuchungsamt Rheinland-Pfalz” according to German federal law §8 Abs. 1 TierSchG (animal protection law), permission numbers 177-07/G09-1-004 and 177-07/G14-1-015.

## Author Contributions

RH, NL, and MR designed the work, analyzed and interpreted the data, and drafted the work. RH, JP, and SE conducted the work and analyzed the data; JP and SE revised the manuscript critically for important intellectual content.

## Conflict of Interest Statement

The authors declare that the research was conducted in the absence of any commercial or financial relationships that could be construed as a potential conflict of interest.
